# P40 Kitchen sponges—home sweet home for carbapenem-resistant *Klebsiella* spp.

**DOI:** 10.1093/jacamr/dlac004.039

**Published:** 2022-02-16

**Authors:** H. Debergh, L. Delbrassinne, C. Garcia-Graells, K. Van Hoorde

**Affiliations:** Sciensano, Belgium; Sciensano, Belgium; Sciensano, Belgium; Sciensano, Belgium

## Abstract

**Background:**

Although research on foodborne diseases is well documented for certain microorganisms, less research has been undertaken on cross-contamination with Enterobacteriaceae from food within the kitchen environment. In this study, we analysed the presence of *Klebsiella* spp. and *Raoultella* spp. and their antibiotic resistance in 100 used kitchen sponges. In a domestic environment, kitchen sponges are commonly used by consumers for doing the dishes and/or cleaning kitchen surfaces. Given their multipurpose use, their often high humidity and presence of organic residuals they are considered a favourable habitat for various groups of microorganisms and could serve as a vehicle in transmission of foodborne pathogens.^1,2^

**Material and methods:**

A total of 100 kitchen sponges alongside a questionnaire regarding hygienic parameters were randomly collected from domestic environments and analysed within 24 h after arrival in the lab. Kitchen sponges were immersed in 100 mL buffered peptone water, homogenized and incubated at 37°C for 24 h ± 2 h. Ten microlitres of this enrichment was plated on MacConkey agar (Bio-Rad) and incubated at 37°C for 24 h ± 2 h. Presumptive *Klebsiella* spp. and *Raoultella* spp. were isolated and confirmed by MALDI-TOF MS. Antibiotic resistance testing (ART) was performed on all *Klebsiella* spp. and *Raoultella* spp. isolates following the EUCAST guidelines. WGS was performed using the Illumina platform.

**Results:**

A total of 65% of the kitchen sponges were positive for *Klebsiella* spp. or *Raoultella* spp. The species *Klebsiella oxytoca* was detected in 78.5% of positive samples, followed by *Klebsiella pneumoniae* (12.31%), *Raoultella ornithinolytica* (4.62%), *Klebsiella variicola* (1.54%), *Klebsiella aerogenes* (1.54%) and *Raoultella planticola* (1.54%). To the best of our knowledge this is the first study performed on detection of *Klebsiella* spp. in kitchen sponges from Belgium and has comparable results with previous studies.^1–3^ We hypothesize the high positivity rate is caused by cross-contamination. ART revealed the presence of one carbapenem-resistant isolate, with resistance to ertapenem and meropenem. Reduced susceptibility to carbapenems was caused by the presence of *bla*_SHV-36_ combined with porin deficiency. *Klebsiella* spp. can be part of the commensal flora of human intestines but are considered to be opportunistic pathogens. *Klebsiella* spp. might present antimicrobial resistance and carbapenem-resistant Enterobacteriaceae are taken up in the WHO global priority list of antibiotic-resistant bacteria.

**Conclusions:**

High prevalence of *Klebsiella* spp. and a carbapenem-resistant isolate in kitchen sponges highlight the occurrence of cross-contamination from food and the possible risks for foodborne disease associated with this kitchen tool. These findings advocate for good hygienic measures within a household setting.
